# Prevalence of HBV in HIV Patients Referred to Imam Khomeini Hospital, Tehran, Iran from 2008-2010

**DOI:** 10.5812/ircmj.4883

**Published:** 2013-04-05

**Authors:** Esmaeil Mohammad Nejad, Seyyedeh Roghayeh Ehsani, Narmela Rabirad, Roghayeh Deljo, Simin Ranjbarn, Shadi Rezaee, Zahra Tamizi

**Affiliations:** 1 Department of Nursing, International Branch, Shahid Beheshti University of Medical Sciences, Tehran, IR Iran; 2 Imam Khomeini Clinical and Hospital Complex, Tehran University of Medical Sciences, Tehran, IR Iran; 3 Department of Hospital Infections, Tehran University of Medical Sciences, Tehran, IR Iran; 4 Department of Hospital Infection Control, Farabi Hospital, Tehran University of Medical Sciences, Tehran, IR Iran; 5 Department of Hospital Infection Control, Razi Hospital, University of Social Welfare and Rehabilitation, Tehran, IR Iran

**Keywords:** HIV, HBV, Coinfection, Iran

*Dear Editor,*

Human immunodeficiency Virus (HIV) and hepatitis B are prevalent and important viral infectious throughout the world and are considered as an important problem (1) HIV related immunosuppressive increases significantly the risk of acquiring opportunistic infections due to hepatitis B. The opportunistic infection is a major source of mortality and mobility in HIV-related patients. Globally an estimated 350-400 million people are chronically infected with HBV and 33 million are living with HIV infection today (2). In Iran, it was estimated that HIV cases are approximately 22000 to 30000 and over 35% of the Iranians have been exposed to HBV, about 3% are chronic carries and its frequency ranges from 2 to 3 percent (3), despite of known efficacy of highly Active Antiretroviral Therapy (HAART) of HIV infected patients (4). Various international studies have been conducted to demonstrate the rate of co-infection with HBV and the result are naturally sought according to subpopulation and country (5). Recently, hepatitis B co-infection was associated with a poor overall survival in patients with HIV (6). On the other hand, few studies investigated the chronic HBV prevalence and correlated factors among HIV patients. The aim of this study was to determine seroprevalence of HBV infection and associated risk factors among HIV patients referral to Imam Khomeini hospital of Tehran, the capital of Iran. This was a cross-sectional study which was done on 213 patients with HIV referred to Imam Khomeini Hospital Complex at Tehran University of Medical Sciences for evaluation of HBV serologic markers from October 2008 to October 2010. Our samples were all of HIV patients who referred to Imam Khomeini hospital after getting the informed consent data sheet completed by interviews. Data included (gender, education, occupation, marital status), clinical characteristics (CD4 count through flocymetry, opportunistic infection, antiretroviral treatment), risk behavior pattern (blood transfusion, alcohol consumption, high risk sexual activities, Intravenous Drug User (IDU)). The serum sample 5 cc of venous blood from confirmed HIV positive patients were measured by commercially available Enzyme Linked Immunosorbet Assay (ELISA) and the HBsAg kit (Biokite Spanish). Participation in the study was done voluntarily after obtaining informed consent. We used mean + SD (standard deviation) or proportions for continuous or categorical variables. Independent risk factor for HBV infection was assessed using multivariate logistic regression model. P values of 0.05 or less were considered statistically significant. All statistical analyses were performed using Statistical Package for Social Sciences Software (SPSS, version, 16). Two hundred thirteen HIV infected patients had referred to Imam khomeini hospital from 2008 to 2010 .The range of HIV patients’ age was between 16-58 old years with average of 35 + 8/1. Their mean CD4 count was 202.9 + 9.5 cell/ml. Our samples were 91.5% male and 8.5% female. The seroprevalence of HBS Ag among 213 HIV/AIDS patients was 11.2%. CD4 counts (cells/ml) were categorized into 3 groups: CD4 < 200, CD4 count 200-499 and CD4 > 500 (cells/ml). Majority of patients had CD4 count 200-499 cell/ml. Mean of CD4 count in HIV/HBV were 207.89 + 29.21. Most common risk factors for HIV infection were injection, drug use (46. 4%) and sexual transmission (28.6%) (Table 1).There was significant relationship only between seroprevalence in case of HBs Ag and HIV drug users (P = 0.001).

**Table 1.** Risk factors of Patients for HIV Infection

Risk Factors, No. (%)	
**Injection Drug Use (IDU)**	99 (46.4)
**Sexual contact**	61 (28.6)
**Blood product**	6 (2.8)
**Unknown**	12 (5.6)
**Multipel Risk Factor**	35 (16.4)

In our study, it is indicated that the average prevalence of HBV among HIV infected patients was 11.3 %, Rahimi-Movaghar et al. indicated that HBV infection among those infected with HIV reached to 7.8% (7). Also, according to Zago and her Colleagues, the overall estimated HBV prevalence among patients infected with HIV was 3.8% (7). Our present study indicates that the frequency of HBSAg was significant in IV drug users. Drug user was an important route of transmission of HBV. Dimitrakopoulos et al. found a higher frequency of HBSAg among IV drug users than among homo/bisexuals and also, the prevalence of HBV markers in that group was 67.4%: 71.8% in homo/bisexuals, 35.3% in heterosexuals, 91.7% in IDUs and 90.9% in blood transfusion recipients (8). Alavi et al. reported the co-infections of HBV in 104 HIV positive drug addicts who were hospitalized in the infectious ward between 2001-2003 in Razi hospital, Ahvaz, Iran, it was estimated to 44.35% (9). The results of this research showed that only Intravenous drug abuse (IVDA) had a significant risk factor for HBV/HIV. In a similar study performed on 130 HIV positive patients in Iran, none of these patients showed significant difference for co-infection with HIV/HBV (10). According to these results, it is defined that IDU is the highest risk factor for acquisition of HBV/HIV infections. We recommend screening for HBSAg positive in HIV infected patients, especially for patients with high risk behaviors.
